# The Legislature and a Board of Examiners

**Published:** 1893-11

**Authors:** 


### THE LEGISLATURE AND A BOARD OF EXAMINERS.

The Legislature has assembled and by this time has gotten fairly to work. The matter of the examining board will come up during this session, and the friends of protective legislation in the State hope very much that it will receive favorable action. The Legislature cannot argue any more that it has not had time to give this matter an intelligent and thorough consideration. The members have been made acquainted with the objects sought after and with the arguments sustaining the same. With any intelligent body of men that are at liberty to act according to their judgment, we do not believe that these arguments can fail to be appreciated. They are simply irresistible. The State Medical Association has indorsed the movement, and petitions the Legislature to establish the board.

The better part of the profession generally recognizes the necessity of some system of examining those persons who wish to practice medicine in the State. It is only an act of justice to the masses of people in the State that the Legislature furnish them with the protection which goes with an efficient examining board. The number of quacks, medical impostors and frauds that conduct their shameful business in Georgia is becoming greater and greater every year. Why? Because our gates stand open wide to all who choose to enter, regardless of qualifications, and the great army of charlatans and incompetents find in Georgia the homes which are denied them in other States. About thirty States and one or two Territories are now operating these boards in one form or another, and it is the universal verdict that they exert an influence that is highly potent for good. Those that oppose them are generally those who are benefited in one way or another by the loose conditions which exist wherever they are absent.

We hope our subscribers throughout the State will use their influence upon their representatives in the Legislature, urging them to support this bill. In the language of a friend who wrote us about it some time ago, "*There is every need for it, God knows.*"

